# The Impact of Early Palliative Care Decisions on Hospital Service Utilization and End-of-Life Care in Patients with Pancreatic Cancer—A Retrospective Study

**DOI:** 10.1177/08258597261436077

**Published:** 2026-03-29

**Authors:** Sofia Koivusalo, Pauliina Kitti, Nelli-Sofia Nåhls, Timo Carpen, Riikka-Leena Leskelä, Tiina Saarto, Outi Akrén

**Affiliations:** 1 Palliative Center, 60652 Turku University Hospital and University of Turku, Turku Finland; 2 Department of Radiotherapy, Comprehensive Cancer Center, 3836 Helsinki University Hospital and University of Helsinki, Helsinki, Finland; 3 Department of Oncology, 60656 Vaasa Central Hospital, The Wellbeing Services County of Ostrobothnia, Vaasa, Finland; 4 Palliative Care Center, Comprehensive Cancer Center, Helsinki University Hospital, and Faculty of Medicine, 159841 University of Helsinki, Helsinki, Finland; 5 629600 Nordic Healthcare Group, Helsinki, Finland

**Keywords:** pancreatic cancer, palliative care, end-of-life care, health care utilization

## Abstract

**Objectives:**

Early palliative care (PC) is recommended in pancreatic cancer but remains underutilized*.* This study assessed whether the timing of the PC decision affected the hospital resource use and access to specialized PC services. The implementation of an integrated PC (IPC) was also evaluated.

**Methods:**

This retrospective single-center cohort study included 440 deceased pancreatic cancer patients treated at the Comprehensive Cancer Center, Helsinki University Hospital (2017–2018). Patients were categorized by timing of the PC decision—defined as withholding or termination of life-prolonging treatment and transition to PC—into early (>30 days before death) or late/no (≤30 days before death) groups. Hospital resource utilization was obtained from electronic medical records.

**Results:**

A PC decision was made for 87% of patients, median of 1.5 months before death. Chemotherapy was given to 8% during the last month. Compared to early decisions, late/no PC decisions were associated with anticancer treatment closer to death (43 days vs 115 days, p < 0.001), higher acute healthcare use, including double the emergency department visits (61% vs 27%, p < 0.001) and triple the hospitalizations (59% vs 20%, p < 0.001) in the final month. Early PC decision was associated with earlier and more frequent use of the outpatient PC unit (3.6 vs 1 month before death, p < 0.001; 84% vs 61%, p < 0.001). Only 36% received PC integrated with oncologic treatment.

**Conclusions:**

Late or absent PC decisions were associated with increased end-of-life hospital interventions and reduced access to specialized PC services; both linked to impaired quality of EOL care and increased healthcare costs.

## Introduction

Pancreatic cancer is one of the most aggressive malignancies with poor prognosis. Despite achievements in cancer care, the 5-year overall survival of pancreatic cancer has barely changed over the past decades, remaining at 5%–12%.^1^ In Finland, approximately 1300 new pancreatic cancer diagnoses are made each year.^2^ Globally, Globocan reports 510,992 new cases with incidence and mortality rates rising worldwide.^3^ Most of the cases are beyond curative care at diagnosis and the goal of the care is disease modifying or stabilising to extend life expectancy. For 25%–50% of the patients, the intent of care is solely palliative already at the time of the diagnosis.^4–6^ Life-prolonging anticancer therapy in first and second line is recommended for patients with an adequate performance status. The proportion of patients receiving a third line of chemotherapy is increasing,^7^ even though its efficacy is controversial, nor is it recommended.^8,9^ Furthermore, indicators of a more aggressive end-of-life (EOL) care have been noticed in previous studies concerning patients with advanced cancer.^10,11^ Integrated palliative care (IPC), introduced alongside anticancer treatment, has been shown to reduce aggressive EOL care, lower hospitalization rates, and improve quality of life (QoL).^12,13^ Given the poor prognosis of pancreatic cancer, the need for IPC is especially critical. Despite these benefits and guideline recommendations, the proportion of pancreatic cancer patients receiving IPC remains low, at approximately 30% in several studies.^14–17^

Previous Finnish studies have shown that delayed or absent PC decisions lead to more aggressive EOL care, while early PC intervention and PC decision reduce hospitalizations, emergency department (ED) visits, and healthcare costs.^18–20^ Aggressive treatments and unplanned care near death are considered indicators of poor quality EOL care.^21^

In our previous single-center study of pancreatic cancer patients (2013–2014), an early PC decision—defined as terminating life-prolonging treatment more than 30 days before death—was documented in 56% of cases and was associated with fewer ED visits and hospitalizations in the last month of life.^22^ To further explore strategies for reducing burdensome EOL care, we conducted a new retrospective cohort study, focusing on the association between PC decision timing and the use of healthcare services (ED visits and hospitalizations) in patients with pancreatic cancer. Additionally, we examined the extent to which integrated palliative care was implemented in clinical practice.

## Methods

### Study Design

This retrospective cohort study included adult patients diagnosed with pancreatic cancer.

### Setting and Participants

The retrospective study cohort (n = 440) comprised all adult patients with pancreatic cancer diagnosis (ICD-10 code C25) who had been treated at the Comprehensive Cancer Center Helsinki University Hospital (HUH) in 2017-2018 and were deceased by the end of 2018. Patients who died before April 1, 2017 (n = 36) were excluded due to limited follow-up availability. Patient data were obtained from hospital registries.

### Cancer Care and Palliative Care in Finland

In Finland, most patients with cancer are initially evaluated and treated within secondary and tertiary healthcare. HUH is the largest of the country's five tertiary hospitals and provides specialized cancer care for approximately 1.6 million residents in Southern Finland. During the time of this study, the Comprehensive Cancer Center at HUH delivered all radiotherapy and the majority of systemic anticancer treatments for adult patients, with the exception of certain non-chemotherapy treatments provided by urologists and endocrinologists. The Comprehensive Cancer Center includes a specialist palliative care (SPC) unit that provides outpatient services and inpatient consultative support across HUH, although it does not operate a dedicated inpatient PC ward. At the specialized level, PC is provided by healthcare professionals with dedicated training in PC whose clinical practice is focused exclusively on PC patients.

Nationally, PC is integrated within the public healthcare system and extends beyond EOL care, encompassing symptom management and psychosocial support that may also be delivered alongside cancer-directed treatment. In Finland, PC is organized into two levels, general and specialized. SPC includes outpatient clinics, consultation teams, inpatient palliative wards, hospices, and hospital-at-home services.^23,24^ SPC services are delivered across primary, secondary, and tertiary healthcare. Unlike hospice-based systems that rely on prognostic thresholds, the Finnish PC model is guided primarily by intent-of-care decisions rather than estimated survival. This distinction applies to the formal PC decision, which denotes a documented shift in treatment, whereas integrated PC may be introduced earlier alongside cancer-directed treatment based on patient needs, independent of the timing of that decision.

### Palliative Decision, Palliative Care Phase and Specialized Palliative Care

The PC decision was defined as the point at which life-prolonging anticancer treatment was discontinued or not initiated and the intent of care shifted to PC. This decision is made by the oncologist overseeing the patient's care, typically within a multidisciplinary framework, in accordance with national clinical practice and documentation standards. In Finland, the transition is formally documented using the ICD-10 code Z51.5, which reliably marks the timing of a recorded PC decision. The PC phase is defined here as the time from the PC decision to death. Based on the timing of this decision, patients were categorized into two groups: early PC decision (>30 days before death) and late or no PC decision (≤30 days before death or no documented decision). The 30-day cutoff aligns with established EOL quality indicators and enables comparability with prior Finnish and international studies.^19,25,26^ In the late/no PC decision group, time-related analyses were restricted for patients with a documented PC decision.

IPC was defined as a first visit to the Cancer Center's Outpatient SPC unit occurring while the patient was still receiving oncological treatment. IPC reflects timing of SPC contact, rather than timing of discontinuation of anticancer treatment, ie, the PC decision. Therefore, patients may receive IPC despite having a late PC decision. Accordingly, this study focuses on the timing of PC decision-making rather than referral to SPC alone.

### Data Sources and Collection

Data collection included patient demographics, ICD-10 codes, outpatient visits to the Comprehensive Cancer Center HUH, given anticancer therapy, date of the PC decisions, visits to the ED and number of hospitalizations and inpatients days in secondary/tertiary care, time of death and age at death. All data was retrieved in a structured format, except for the date of the PC decisions, which were extracted either from ICD-10 code Z51.5 (palliative care) or, if no diagnosis code was recorded, a mention of PC decision in the free text of the patients’ records. The free text search within the patients’ medical records was done using a few keywords, eg palliative, EOL care. The date of the PC decision was defined as the date when diagnosis Z51.5 was recorded for the first time, or the goal of care was defined as palliative in the free text in the medical records.

### Statistical Analysis

All analyses were performed with IBM SPSS Statistics version 29 (IBM Corp., Armonk, NY, USA). Descriptive statistics for continuous variables are presented as medians, with dispersions reported as either minimum–maximum ranges or interquartile ranges (Q1–Q3), as specified. Categorical variables are presented as numbers and percentages. We compared early versus late PC decisions using Pearson's chi-square or Fisher's exact test for categorical variables. Continuous variables (eg, time intervals) were compared with the Mann-Whitney U test. A p-value < 0.05 was considered statistically significant. Logistic regression analyses were performed to identify independent factors associated with ED visits and hospitalizations during the last 30 days of life.

### Ethical Considerations

The present study is a retrospective register study based on hospital registry data of deceased patients from 2017 to 2018. No human interventions were included in this registry-based, retrospective study of deceased patients. According to Finnish legislation, no ethical approval is needed in retrospective registry studies (Act on the Secondary Use of Social and Health Information 522/2019). Authorities of Helsinki University Hospital have approved the study protocol (HUS/325/2023) and waived the need for ethical approval and for informed consents based on the legislation of retrospective register-based studies (522/2019). We confirm that all methods were carried out in accordance with relevant guidelines and regulations.

## Results

The study cohort consisted of 440 patients with pancreatic cancer, 53% were male. Mean age at death was 72 years (range 29–93 years). A PC decision was done for 87% of patients, at some point of the disease trajectory. The median time in days from the PC decision to death, ie the duration of palliative phase was 45 days. The characteristics of patients according to the timing of the PC decision are summarized in Table 1.

**Table 1.** Patient Characteristics According to Timing of the Palliative Care Decision.

	Total n = 440 (%)	Early PC Decision >30 days before death (n = 235, 53%)	Late/no PC Decision ≤ 30 days before death (n = 205, 47%)	P-Value
Age at death median (range)	72 (29-93)	75 (29-93)	71 (52-93)	<0.001
Sex				<0.001
Male	231 (53%)	104 (44%)	127 (62%)	
Female	209 (48%)	131 (56%)	78 (38%)	
PC decision	384 (87%)	235 (100%)	149 (72%)	<0.001

Abbreviations: PC = Palliative care, n = number of patients

### Integrated Palliative Care

IPC, defined as SPC contact during ongoing anticancer treatment, was implemented in just over one third of the cohort (Table 2). When IPC was combined with an early PC decision, patients experienced a notable longer PC phase (median length of 2.8 months vs 0.5 months, respectively) and earlier initiation of SPC contact (a median 6.1 months vs 2.2 months prior to death, respectively) compared with early PC decision without IPC.

**Table 2.** The Timing of Palliative Care Decision and SPC Outpatient Unit Visits.

	Total n = 440 (%)	Early PC Decision >30 days before death (n = 235)	Late/no PC Decision ≤ 30 days before death (n = 205)	P-Value
Cancer Center's Outpatient PC unit visit	323 (73%)	198 (84%)	125 (61%)	<0.001
Median time (days) from the first visit to the Cancer Center's Outpatient PC unit to death (Q1-Q3)	77 (36-169)	108 (66-199)	30 (18-77)	<0.001
Median length of palliative phase ie time in days from PC decision to death (Q1-Q3)	45 (18-100)	79 (53-138)	13 (6-21)	<0.001
IPC	160 (36%)	95 (40%)	65 (32%)	0.060
Median time (days) from a first visit to the Cancer Center's Outpatient PC unit to death (range) in patients with IPC (n = 160/440)	112 (6-593)	183 (36-593)	65 (6-463)	<0.001
Median length of palliative phase ie time in days from PC decision to death (Q1-Q3) in patients with IPC	52 (23-105)	85 (52-138)	15 (5-24)	<0.001

Abbreviation: PC = Palliative care, IPC = Integrated palliative care, n = number of patients

Footnote: Continuous variables are presented as median with minimum–maximum range or with interquartile range (Q1–Q3).

### Health Care Utilization and Anticancer Therapy During the Last Month of Life

Patients with late or no PC decisions had a substantially higher probability of acute health care utilization, both ED visits and hospitalizations, during the last month of life, compared with patients who had an early PC decision (Table 3).

**Table 3.** Health Care Utilization and Use of Anticancer Therapies at EOL According to the Timing of the Palliative Care Decision.

	Total n = 440 (%)	Early PC Decision >30 days before death (n = 235)	Late/no PC Decision ≤ 30 days before death (n = 205)	P-Value
Emergency department visits within 30 days before death	188 (43%)	64 (27%)	124 (61%)	<0.001
Hospitalizations within 30 days before death	168 (38%)	47 (20%)	121 (59%)	<0.001
Inpatient days (median, range)	6 (1-30)	4 (1-19)	7 (1-30)	<0.001
Radiotherapy in the last month of life	16 (4%)	6 (3%)	10 (5%)	0.194
Median time in days from last radiotherapy to death (range)	65 (2-636)	75 (6-509)	36 (2-636)	<0.059
Chemotherapy in the last month of life	37 (8%)	<3 (0%)/NA	36 (18%)	<0.001
Median time in days from last chemotherapy to death (range)	75 (6-575)	115 (30-454)	43 (6-575)	<0.001

Footnote: Continuous variables are presented as median with minimum–maximum range

Continuation of disease-modifying anticancer therapy into the last month of life was relatively rare in this cohort, occurring in fewer than 10% of patients. It was more likely among patients with a late or no PC decision, as patients with an early PC decision were unlikely to receive any anticancer therapy during the final month of life. Patterns of healthcare utilization and anticancer treatment according to the PC decision are presented in Table 3.

In multivariable logistic regression analysis, a late PC decision was strongly associated with increased acute healthcare utilization near the end of life. Compared with an early PC decision, a late decision was associated with a more than fourfold increase in the odds of ED (OR 4.12, 95% CI 2.70–6.29, p < 0.001) and a more than a fivefold increase in the odds of hospitalizations (OR 5.28, 95% CI 3.40–8.22, p < 0.001). Age at death, sex, and visits to a PC unit were not significantly associated with either outcome. Regression results are presented in Table 4.

**Table 4.** Factors Associated with Acute Care Utilization in the Last 30 Days of Life: Logistic Regression Analysis.

	OR	95% CI	p-Value
A. Emergency department visits			
Age at death	0.991	0.970–1.013	0.431
Sex (Ref. male)	1.026	0.679–1.550	0.904
Palliative care decision (Ref. early)	4.120	2.698–6.292	<0.001
Palliative care unit visit	1.089	0.683–1.737	0.720
B. Hospitalizations			
Age at death	0.982	0.960–1.005	0.124
Sex (Ref. male)	0.819	0.532–1.261	0.364
Palliative care decision (Ref. early)	5.283	3.398–8.215	<0.001
Palliative care unit visit	0.883	0.547–1.424	0.609

## Discussion

In this single-center, retrospective study, we observed that delayed or absent PC decisions in patients with pancreatic cancer were associated with a higher probability of acute hospital services during the last month of life, including ED visits, hospitalizations, and continuation of anticancer treatments near death. This study focuses on the timing of PC decision-making rather than referral to PC services alone.

### Palliative Care Decisions

While PC extends beyond EOL care, the PC decision operationalizes a transition in treatment intent rather than the full scope of PC delivery. The Finnish PC model is primarily guided by intent-of-care decisions, which may influence both the timing and patterns of PC integration observed in this cohort. Most patients (87%) had a documented PC decision, made a median of 1.5 months before death. This aligns with a previous Finnish study of tertiary hospital cancer center population,^19,27^ which reported that 82% of cancer patients had a PC decision. However, in that study, the decisions were made somewhat earlier, with 67% occurring within the last 3 months of life. This difference is probably due to the inclusion of all cancer types, including those with less aggressive disease courses than pancreatic cancer. In another national study with 2000 cancer patients, 75% had a PC decision.^27^

### Anticancer Therapies at EOL

In this cohort, only 8% received anticancer treatment during the last month of life, reflecting a conservative approach consistent with guidelines discouraging aggressive EOL care.^28^ This is comparable to previous findings, with reported proportions of cancer treatment in the final month ranging from 2% to 23%.^15,29,30^ In our study, anticancer therapies were often discontinued well before death (median 75 days), yet the formal PC decision occurred only 45 days prior to death, indicating a late transition to the PC phase and limited time to ensure quality EOL care for most patients.

Our findings showed that delayed or absent PC decisions were associated with anticancer treatment being continued closer to death. This aligns with previous research indicating that late initiation of PC in pancreatic cancer can lead to prolonged aggressive anticancer therapy, sometimes up until the final weeks or days of life.^15,31^ This highlights the importance of timely initiation of the palliative phase to avoid potentially non-beneficial continuation of anticancer treatment near death. Furthermore, studies suggest that patients under the care of oncologists during the final month are more likely to receive late-stage chemotherapy, reducing the duration of PC support and increasing the risk of treatment-related harm.^7,32–34^

### Contact to the Palliative Care Unit

Most of this cohort (73%) received SPC. Compared to our previous study from the same cancer center (2013–2014),^22^ where 49% of pancreatic cancer patients accessed SPC services, the current findings indicate a clear improvement. International studies have shown lower rates of PC referrals. In a large cohort study of 54,130 pancreatic cancer patients, only 6% accessed PC services.^35^ Other studies report referral rates of 30%–50% in this patient population.^14,15,29^

### Timing of Palliative Unit Contact and Palliative Care Phase

While most patients had both a PC decision and access to SPC, both were often implemented relatively late in the disease trajectory. The median timing of the first contact with the Cancer Center's Outpatient PC unit was 2.6 months before death. In line with the previous studies, patients with early PC decisions were more likely to be referred to the PC unit, and to do so earlier, compared to those with a late or no PC decision. PC decision and referral to specialized palliative care seemed to interrelate.

Late referrals to the PC unit occurring only after interruption of anticancer therapies have also been shown in a previous national study^27^ as well as internationally, with up to 70% of pancreatic cancer patients referred within the final month of life.^35^ Despite variability in the definition of late referrals, their prevalence is consistently reported in several studies.^14–17^^,35^ Similar patterns are observed across other advanced malignancies.^31,36^ The timing of the first PC unit contact coincides with the termination of disease-modifying therapy, rather than at an earlier point of the disease course. In line with the previous studies, one-third of the patients in this cohort had their first visit to the PC unit occurred while still receiving anticancer treatments, representing IPC. Nevertheless, IPC was initiated late (median 3.7 months before death). Only 22% of patients, those with both IPC and an early PC decision, received PC aligned with current oncology societies’ recommendations,^37,38^ which emphasize timely initiation of PC for patients with advanced malignancies, including pancreatic cancer. In this subgroup, the median duration of the palliative phase was approximately 3 months, and the time from the initial PC unit referral to death was 6 months. These findings reinforce the importance of integrating PC earlier in the disease trajectory, as late PC decisions leave insufficient time for meaningful PC engagement.

### Utilization of Hospital Services

We found that the timing of the PC decision was significantly associated to healthcare resource utilization. A delayed or absent PC decision was associated with increased use of acute hospital services during the final month of life. Patients with late or no PC decisions had twice the number of ED visits and three times the hospitalization rate compared to those with an early decision. Previous studies have shown that earlier PC referrals (30-90 days before death) in patients with pancreatic cancer are associated with fewer ED visits and hospitalizations in the last month of life.^14,15,26^ However, the timing of SPC referrals remains unclear. While some studies report reductions in aggressive EOL care in regard to timing of referral,^30^ others have found no consistent association between timing of PC referrals and health care utilization.^14,35^ Some studies report higher healthcare utilization among patients with pancreatic cancer receiving PC, which likely reflects both the aggressiveness of the disease and the need for intensive symptom management near the EOL.^29,35^ It appears that both an early PC decision and SPC contact act synergistically, with their combination resulting in the greatest reduction in acute hospital service use, as reported by Hirvonen et al^19^ The reduction in healthcare utilization observed following a PC decision should not be interpreted as abandonment of symptom-directed or clinically indicated treatment. Within the Finnish care model, symptom-relieving interventions—including palliative radiotherapy and, in selected cases, surgical procedures—may still be provided after a PC decision. Lower use of acute hospital services therefore more likely reflects a shift in treatment goals, improved symptom control, and care delivered in more appropriate settings rather than undertreatment. Given the retrospective nature of this study, the observed associations are non-causal and likely influenced by underlying prognosis, disease burden, and performance status.

Our findings differ from those reported by Zhang et al,^29^ who observed higher rates of chemotherapy use and aggressive EOL interventions among patients receiving PC consultation. These contrasting results likely stem from methodological and contextual differences. Zhang et al examined referral-based PC consultation and healthcare utilization over the final 6 months of life, whereas our study focuses on the timing of a formal PC decision and utilization during the last month of life. Additionally, in Finland the PC decision represents a documented transition in treatment intent, whereas PC consultation in other settings may occur without a corresponding shift in care goals. Notably, Zhang et al also suggested that increased use of aggressive interventions near the EOL may reflect the need for specialty-level symptom management in advanced pancreatic cancer, rather than inappropriate or excessive care.

### Strengths and Limitations

The study has some limitations related to its retrospective, registry-based design. This study was not designed to assess survival outcomes, and the timing of PC decisions should not be interpreted as influencing survival, but rather as reflecting underlying prognosis and disease trajectory. Because clinical variables, such as performance status and comorbidity burden, were not available in structured form, multivariable adjustment for potential confounding was not feasible. Consequently, the observed associations should be interpreted with caution and cannot be considered causal. Further constraints due to data availability are that information on time of cancer diagnosis, cancer stage at diagnosis, and initiation of anticancer treatment could not be extracted. Therefore, analyses could not account for disease trajectory or stage specific differences, which may have affected both the timing of PC decisions and healthcare utilization patterns. By focusing exclusively on deceased patients, the study ensured complete data on EOL care utilization but might have introduced survivorship bias, excluding individuals who survived beyond the study period. Additionally, the study includes data only on PC contacts within tertiary healthcare, which may underestimate the true proportion of patients receiving SPC, as some may have been referred to PC services within primary care. Another consideration is that the data is relatively dated. However, given that the systematic integration of early PC is still not standard practice everywhere, the findings may still accurately reflect current conditions in various clinics. The strength of the study is its population-based real-world design, which included all patients with pancreatic cancer treated in the Cancer Center over a defined period, giving a rather realistic and comprehensive overview of the care provided to this patient group during the study interval. Furthermore, the information on hospital service utilization is largely comprehensive and reliable.

This study adds to the growing evidence that the timing of PC plays a critical role in shaping EOL care trajectories for patients with pancreatic cancer. Clinically, an earlier PC decision may serve as a meaningful process-of-care quality marker, reflecting timely recognition of patient needs and alignment of treatment goals as the disease progresses. From a health system perspective, earlier PC decisions were associated with a lower likelihood of acute care utilization near death, suggesting a reduced burden on emergency and inpatient services and improved coordination between oncology teams and SPC services. Further studies are required to separate the influence of prognosis, disease severity, and clinician decision-making from the timing of PC initiation, in order to better elucidate the causal pathways through which palliative care integration affects healthcare utilization and the quality of EOL care.

## Conclusions

A timely PC decision is essential to initiate the PC phase, as delays increase the likelihood of aggressive EOL care and reduce access to specialized PC services.
